# Antipsychotic medication for behaviors that challenge in individuals with intellectual disabilities: a clinically informed review

**DOI:** 10.3389/fpsyt.2025.1609408

**Published:** 2025-07-28

**Authors:** Alessandro Pascucci, Fabienne Gerber, Marie Besson, Markus Kosel

**Affiliations:** ^1^ Psychiatry Department, Geneva University Hospital, Geneva, Switzerland; ^2^ Psychiatry Department, Faculty of Medicine, University of Geneva, Geneva, Switzerland; ^3^ Division of clinical pharmacology and toxicology, Geneva University Hospital, Geneva, Switzerland; ^4^ Department of Anesthesiology, pharmacology, Intensive care and Emergency Medicine, University of Geneva, Geneva, Switzerland

**Keywords:** intellectual disabilities, pharmacological interventions, antipsychotics, risperidone, olanzapine, aripiprazole, zuclopenthixol, challenging behaviors

## Abstract

Individuals with intellectual disabilities (ID) frequently exhibit behaviors that challenge (BC), such as aggression and self-injury, which significantly impact their quality of life. Pharmacological interventions, particularly antipsychotics, are regularly employed to manage these behaviors. However, these medications are frequently prescribed off-label, increasing the risks of polypharmacy, drug-drug interactions, and potential adverse effects. We conducted a comprehensive literature search to identify studies on antipsychotic interventions for BC in individuals with ID. Eligible studies included observational (cross-sectional and longitudinal) studies and randomized controlled trials (RCTs). Findings from RCTs were mixed: while some trials reported reductions in aggression and irritability with antipsychotics such as risperidone and olanzapine, others showed no advantage over placebo or supported deprescription strategies. Observational studies generally supported the short-term effectiveness of risperidone, olanzapine, and zuclopenthixol in reducing aggressive behaviors, although evidence for their impact on self-injurious behaviors (SIBs) was inconsistent. Across both study types, the use of antipsychotics was consistently associated with adverse effects, including sedation, weight gain, and metabolic changes. Preliminary open-label evidence suggested that aripiprazole may reduce BC in individuals with Fragile X Syndrome (FXS), while causing fewer metabolic side effects. These findings highlight key limitations of the current literature, including the scarcity of studies focusing specifically on ID populations, small sample sizes, the limited number of RCTs, and often controversial or inconsistent results. Despite these limitations, the review indicates potential benefits from reducing dosages and discontinuing long-term antipsychotic use, particularly when guided by personalized treatment plans and regular reassessment. Overall, the results support cautious and individualized prescribing, with close monitoring of adverse effects and attention to deprescribing when appropriate. Further longitudinal and naturalistic studies are warranted, along with the development of structured tools to assist clinicians in optimizing pharmacological care for this vulnerable population.

## 1 Introduction

Individuals with intellectual disabilities (ID) exhibit neurodevelopmental deficits that impair both intellectual functioning and adaptive behavior. ID affects approximately 1% of the overall population (1), making it a relatively common neurodevelopmental disorder. The principal etiologies of ID include genetic conditions (such as Down syndrome and Fragile X Syndrome, FXS), prenatal factors (e.g. fetal alcohol syndrome or infections during pregnancy), perinatal complications (such as oxygen deprivation during birth), and postnatal causes (including traumatic brain injury, severe infections, and exposure to toxins). Additionally, environmental factors such as malnutrition, lack of stimulation, and social deprivation can contribute to the development of ID (2).

The impact of ID reaches beyond those directly affected, as it presents significant challenges for their families and communities as well. This results in considerable socioeconomic consequences, such as higher healthcare costs, special education needs, and additional support services (3).

Behaviors that challenge (BC**),** including irritability, aggression, self-injury, and other disruptive manifestations, pose significant difficulties for individuals with ID (4). These behaviors impact the quality of life of the individuals and their caregivers, necessitating effective intervention strategies. Pharmacological interventions, particularly atypical antipsychotics (5, 6), are routinely used in clinical practice to manage these behaviors (7, 8).

This practice is often in contrast with international (9) and national recommendations, such as National Institute for Health and Care Excellence (NICE) guidelines (10, 11) and 2018 Canadian consensus guidelines (12). These guidelines recommend that antipsychotics should not be used to treat BC in people with ID unless a comorbid psychotic disorder is present or unless other non-pharmacological approaches have been attempted and proven ineffective (13). As well as not being very effective on average in preventing CB, the use of those medications in ID population often involves potentially inappropriate prescriptions (14, 15), leading to polymedication (16) drug-drug interaction issues and adverse drug reactions (17). Despite these recommendations, antipsychotics continue to be frequently prescribed for BC in this population, often outside their officially approved indications. In fact, a substantial proportion of these prescriptions are considered off label. Importantly, “off-label” use in this context can refer to several distinct scenarios — including prescriptions outside approved age ranges, for diagnoses not specifically indicated, or for behavioral symptoms in the absence of a formal psychiatric disorder. Each of these carries different safety and efficacy profiles, which may significantly influence clinical outcomes and raise ethical concerns. Recognizing this heterogeneity is crucial to fully grasp the complexities of antipsychotic use in individuals with ID.

In clinical practice, antipsychotics seem to be partially effective for specific BC such as irritability and aggression (18), but they do not appear as effective for other BC such as self-injurious behaviors (SIBs), repetitive behaviors, and hyperactivity (19, 20).

Due to the significant discrepancy between guideline recommendations and common clinical practices despite the low level of effectiveness, we conducted a literature review to seek robust evidence on the appropriate use of antipsychotics for managing BC in individuals with ID. This review focuses specifically on CBs in individuals with ID, rather than those occurring in the context of autism spectrum disorder (ASD). Although ASD and ID frequently co-occur and may share certain behavioral features, they represent distinct clinical populations with differing neurodevelopmental profiles, diagnostic frameworks, and treatment responses. Including studies primarily focused on ASD would have introduced significant heterogeneity, potentially limiting the applicability of findings to individuals with ID. This distinction is clinically meaningful, as highlighted by Thurm et al. (21), who emphasize the diagnostic and phenotypic divergence between ID and ASD despite overlapping symptoms.

The aim of this comprehensive review was to synthesize existing literature on the efficacy and safety of antipsychotic drugs interventions in this specific patient population, while highlighting potential limitations. By critically examining these findings within the context of clinical practice, we seek to inform clinical decision-making and improve therapeutic approaches.

## 2 Methods

A comprehensive search was conducted on PubMed using Medical Subject Headings (MeSH) terms and keywords to identify relevant studies on antipsychotic pharmacological interventions for individuals with ID and CBs. The search strategy included terms like “Intellectual Disability,” “Drug Therapy,” “Psychopharmacology,” “Antipsychotic Agents,” “Aggression,” “Disruptive Behavior,” “Conduct Disorder,” “Self-Injurious Behavior,” “Hostility,” and “Impulsive Behavior.” An example of the search string used is: (“intellectual disability” AND “antipsychotic agents” AND “aggression”). The search covered studies published from January 1990 to June 2024, focusing on RCTs or cross-sectional or longitudinal observational studies. Review papers, abstracts, and case reports were excluded. Articles were screened based on title and abstract screening, and retained articles were assessed for full text eligibility, leading to the final included articles. This review was conducted in accordance with the PRISMA (Preferred Reporting Items for Systematic Reviews and Meta-Analyses) guidelines (22), providing a standardized framework for identifying and selecting studies. Eligibility criteria for article selection were defined using the PICOS (Population, Intervention, Comparison, Outcomes, Study design) approach (23), as detailed in **Supplementary Table 1**.

## 3 Results

### 3.1 Study selection

We initially identified 221 articles. After title and abstract screening, 22 articles were *selected* for full text assessment. Following full-text screening, 8 studies were *identified* based on their relevance and methodological rigor (see PRISMA flow diagram (22), in **Supplementary Table 2**). The remaining 14 full-text examined studies were excluded since they did not respect the aforementioned inclusion criteria (**Supplementary Table 1**). The 3 reasons for exclusion were: wrong study design (8 studies), wrong intervention (2 studies), wrong population (4 studies).

Eligible studies included RCTs and robust observational studies with a sample size of at least 10 participants. Studies were required to have clearly described and replicable data collection procedures, a detailed account of the intervention including dosage and duration, the use of validated outcome measures relevant to the research question, and evidence of ethical approval and compliance with research standards. To further assess the quality of the included studies, we conducted a formal risk of bias evaluation. The Cochrane Risk of Bias 2.0 (RoB 2.0) tool (24) was used for RCTs, while the ROBINS-I (Risk Of Bias In Non-randomized Studies of Interventions) (25) tool was applied to observational studies. Key domains included randomization, deviations from intended interventions, missing outcome data, measurement of outcomes, and selective reporting. For observational designs, we also considered confounding and participant selection. A full summary of the risk of bias assessments is provided in **Supplementary Table 3**. One additional study (26) was identified through the citations of previously included studies because it met all inclusion criteria but was not found through the initial search strategy.

The summary of the included studies is presented in **Table 1**.

**Table 1:** Details of the included studies.

Study	Type of study	Sample size	Population	Intervention	Dose	Assessment timing/duration	Outcomes	Side effects	Conclusions
Amore et al. (27)	RCT	62	Adults with ID	Olanzapine/ Risperidone	Olanzapine: 20 mg/day; Risperidone: 6 mg/day	4, 8, 12, 16, 20, 24 weeks	Reduction in aggressive behavior	Sedation, weight gain, hyperprolactinemia (risperidone)	Both effective, olanzapine slightly better for verbal aggression
Ruedrich et al. (20)	Retrospective	31	Adults with ID	Risperidone, olanzapine, quetiapine VS typical antipsychotics	Varies by specific medication (adjusted based on change, or lack of change, in counts of aggression and/or SIB)	12 months	Reduction in aggression; limited impact on SIBs	Weight gain	Poor effectiveness on SIBs; highlights need for metabolic monitoring
Janowsky et al. (6)	Retrospective	20	Institutionalized adults with ID	Olanzapine	Mean dose 9.1mg/day	Data collection period 5 years. Initial assessment period 6 months. Long-term assessment period: mean 20.3 months	Reduction in aggression, SIBs, disruptive behaviors. Reduction in Global Behavioral Rating score. Decrease dosage of concurrent conventional antipsychotics	Sedation, weight gain, constipation	Effective in reducing BC
Hellings et al. (28)	Crossover prospective	40	Children, adolescents, and adults with ID	Risperidone	Low dose (mean 2.0 mg/day for adults, mean 1.0 mg/day for children and adolescents). High dose (mean 3.6 mg/day for adults, mean 2.0 mg/day for children and adolescents)	22-week crossover study, then 24 weeks of open maintenance phase	Reduction in irritability and aggressive behavior at ABC-C Irritability subscale scores. Low dose as effective as high doses	Increased appetite, weight gain, sedation, worse with high doses	Low dose effective. Need for weight control interventions
Tyrer et al. (26)	RCT	86	Adults with ID	Risperidone VS Haloperidol VS Placebo	Risperidone: 1–6 mg/day; Haloperidol: 1–6 mg/day; Placebo	baseline, 4 (primary outcome), 12, and 26 weeks	Significant reduction in aggression in all groups; placebo group showed similar or better outcomes compared to antipsychotics	Sedation, weight gain, metabolic changes (Olanzapine); Extrapyramidal symptoms (Risperidone and Haloperidol)	Antipsychotic drugs may not offer any advantage over placebo in managing aggressive BC in adults with ID
Ramerman et al. (5)	RCT	25	Individuals with ID	Risperidone continuation vs discontinuation	Continuation average dosage 1.97mg/day; Discontinuation: ceased	0 and 24 weeks (8 weeks after complete discontinuation)	No significant change in irritability in discontinuation group. Worsening of stereotypical behaviors	Weight gain, metabolic changes worse in continuation group	Discontinuation of long-term risperidone possible without increased irritability and beneficial to reduce weight and metabolic changes
Erickson et al. (29)	Prospective open label	12	Individuals with FXS	Aripiprazole	Mean dose 9.8 mg/day	12 weeks	Significant improvement in irritability	Mild to moderate tiredness, drooling, akathisia	Well-tolerated and effective in reducing irritability
Hässler et al. (30)	Randomized, Double-blind Placebo-controlled Withdrawal	39	Adults with ID	Zuclopenthixol	2–20 mg/day, starting from low dosage and adjusted (mean 11.4mg/day)	6 weeks open treatment, followed by 12-week randomized withdrawal	Increased aggressive behavior in placebo group	Nausea, insomnia, and diarrhea. No significant difference between groups.	Discontinuation of zuclopenthixol leads to an increase in aggressive behavior
Hässler et al. (31)	Open-label	31 (21 CT, 10 DT)	Adults with ID	Zuclopenthixol	2–20 mg/day, starting from low dosage and adjusted	2 years	CT patients showed sustained improvement in aggressive behavior compared to DT patients	No side effects reported for CT patients. Some unspecified adverse events present for DT patients (3.30%)	Low dose of Zuclopenthixol appears to be effective and safe to reduce the rate of aggression in the long term (2 years)

RCT: Randomized Controlled trial, FXS: Fragile X Syndrome, ID: Intellectual Disability, SIBs: Self-Injurious Behaviors, CT: continually treated, DT: discontinuously treated, BC: behaviors that challenge.

#### 3.1.1 Efficacy of atypical antipsychotics compared to typical antipsychotics

In a randomized controlled trial, the effectiveness and safety of switching to atypical antipsychotics (olanzapine and risperidone) following 6 months of typical antipsychotic treatment for aggressive behavior were assessed in individuals with ID (27). They randomly assigned 62 adult patients with severe ID to either olanzapine treatment group (up to 20mg per day) or risperidone group (up to 6mg per day) and performed blind assessments of outcomes at different time-points (4, 8, 12, 20, 24 weeks). The results showed that both olanzapine and risperidone were more effective compared to typical antipsychotics in reducing aggressive behaviors 24 weeks after the switch, as measured with the Overt Aggression Scale (OAS) (32). The number of episodes after six months of olanzapine or risperidone reported to the number of episodes at baseline suggested that both medications were effective in reducing verbal aggression (p<0.0001), olanzapine being even more effective than risperidone (p=0.0029), while both drugs showing similar efficacy in reducing aggression against objects, oneself or and others (p<0.0001). Risperidone was on the other hand superior to olanzapine on the Clinical Global Impression-Severity (CGI) (33).

Comparison of adverse effects between olanzapine and risperidone revealed that sedation was more prevalent with olanzapine than with risperidone. Extrapyramidal effects only occurred in 2 people in the risperidone group. ECG abnormalities were present only in the risperidone group (3.2%). While weight, fasting glucose, lipid profile, renal, and hepatic functions were similar in both groups, a significantly higher proportion of patients on risperidone (48.4%) had elevated prolactin levels compared to those on olanzapine (35.5%).

Ruedrich et al. (20) conducted a retrospective study comparing the average monthly counts of aggression and SIBs for 1 year of treatment with typical antipsychotics with monthly averages for the next 12 months of treatment with atypical antipsychotics. The 31 patients involved were on risperidone (23), olanzapine (7) and quetiapine (1).

During the year-long treatment with atypical antipsychotics, a significant reduction in aggressive behaviors of 30%was observed for subjects presenting aggression alone. However, for patients with both aggression and SIBs the decrease was no more statistically significant and for those having SIBs alone, a small increase was even observed. An average weight gain of 3 kg was observed, but there was little impact on the other parameters of metabolic syndrome.

The study highlighted that while atypical antipsychotics effectively seem to mitigate aggression compared to typical antipsychotics in individuals with ID, they have no impact on SIBs. Moreover, the observed average weight gain raises concerns about their long-term use.

Janowsky et al. (6) explored the efficacy of olanzapine as an add-on treatment to psychotropic medications, in addressing BC among 20 institutionalized adults with ID in substitution of conventional antipsychotics. They conducted a retrospective study by abstracting data from Neuropsychiatric Behavioral Review (NBR) conference reports. These reports focused on evaluating the treatment response of individuals receiving medications for BC. The study showed significant reduction of cumulative number of target behaviors (aggression, self-injury, and disruptive behaviors) following 6 months of olanzapine treatment (mean dose 9.1mg/day, range 2.5-22.5mg/day). They also measured the Global Behavioral Rating based on NBR, using a 1 to 7 scale (ranging from no maladaptive behavior to severe maladaptive behavior). Ratings were recorded 6 months before starting olanzapine, immediately before starting olanzapine, 6 months after starting olanzapine, and at the end of the study. The mean global rating score dropped by 30% from just before olanzapine was started to 6 months later (p<0.0008). Concurrently, there was a notable decrease in the use of conventional antipsychotic medications within the initial 6 months of olanzapine therapy. Of the 18 subjects taking atypical antipsychotics prior to starting olanzapine, 12 received a lower dose after olanzapine was begun and 5 completely stopped assuming atypical antipsychotics. The decrease in chlorpromazine equivalents comparing before and 6 months after therapy began reached statistical significance. On the counter side a significant increase in weight (mean weight gain of 3.4kg) occurred in the subject group after the first 6 months of olanzapine treatment (p <0.006). Sedation and constipation were the other common side effects noted. This study highlighted the potential efficacy of olanzapine as an add-on treatment for managing BC in institutionalized adults with ID, demonstrating significant reductions in aggression, self-injury, and disruptive behaviors. However, the study also pointed out notable side effects, such as significant weight gain, sedation, and constipation.

#### 3.1.2 Controversy over the efficacy of risperidone

A 22-week crossover study followed by a 24-week open-label maintenance phase was conducted by Hellings et al. (28) to evaluate the long-term effectiveness and tolerability of risperidone in managing irritability and aggressive behavior in 40 children, adolescents, and adults with ID. Participants receiving risperidone were blindly divided into two groups during the crossover phases: one group underwent an initial acute phase 1 with low-dose treatment (mean 2.0 mg/day for adults, 1.0 mg/day for children/adolescents), and then an acute phase 2 with high-dose treatment (mean 3.6 mg/day for adults, 2.0 mg/day for children/adolescents). The second group followed the opposite sequence, starting with high-dose treatment in acute phase 1 and then transitioning to low-dose treatment in acute phase 2.

The study primarily aimed to assess response rates based on the Aberrant Behavior Checklist-Community (ABC-C) Irritability subscale (34).

The findings indicated that 57.5% of participants were full responders, showing a 50% reduction in the ABC-C Irritability subscale scores, while 87.5% achieved a partial response, defined as a 25% reduction in scores. The mean Irritability subscale score decreased from 19.16 (± 9.96) in the placebo group to 11.15 (± 9.28) in the low-dose phase and 13.31 (± 8.92) in the high-dose phase. The mean ABC-C Irritability scores across both drug phases were significantly different from the placebo (p = 0.0002).

Side effects like significant weight gain were common, affecting 70% of subjects. The mean weight gain over the 46 weeks of the study was 7.9 kg for children, 8.3 kg for adolescents, and 6.0 kg for adults. Sedation and gastrointestinal issues were also noted, leading some subjects to drop out of the study.

Notably, the low-dose group was found to be as effective as the high-dose group in reducing irritability and aggressive behavior (no significant statistical difference, p = 0.13). Moreover, the low-dose phase was associated with fewer and less severe side effects.

These results suggest that a low-dose regimen of risperidone offers an optimal balance between efficacy and tolerability.

In a randomized controlled trial, Tyrer et al. (26) compared risperidone, haloperidol, and placebo for treating aggressive BC in adults with ID. The study involved 86 participants, divided into three groups: 28 received haloperidol, 29 received risperidone, and 29 received placebo. Risperidone and haloperidol doses were between 1–6 mg/day. All groups, including the placebo group, showed significant reductions in aggression by the 4-week mark as assessed by the Modified Overt Aggression Scale (MOAS) (35) The median decrease in MOAS scores was 9 for the placebo group (79% reduction from baseline), 7 for the risperidone group (58% reduction), and 6.5 for the haloperidol group (65% reduction).

Secondary outcomes, including the Aberrant Behavior Checklist (ABC score) (36) and the Udvalg for Kliniske Undersogelser (Danish: Committee for Clinical Researches) (UKU) scale (37) for adverse effects, also showed no significant differences between the treatment groups. These findings suggest that antipsychotic drugs may not offer any advantage over placebo in managing aggressive challenging behavior, highlighting the potential for non-pharmacological approaches to be equally effective without the associated side effects.

As the primary endpoint was evaluated at 1 month rather than 3 or 6, we might wonder whether the time taken to observe efficacy was a little too short and whether the placebo effect was particularly present during the first month of treatment.

#### 3.1.3 Effects of long-term treatment

To determine the need for long-term antipsychotic treatment (5), conducted a placebo-controlled, double-blind, randomized discontinuation trial to compare ongoing risperidone treatment versus controlled gradual discontinuation in individuals with ID exhibiting BC, who were on risperidone for at least a year. The primary outcome of the study was measuring changes in irritability assessed by the irritability subscale of the ABC. Among the 25 participants, 11 were randomly allocated to the discontinuation group of whom 82% successfully withdrew from risperidone without a significant increase in irritability compared to the continuation group (p=0.392). However, a significant group-by-time interaction regarding ABC Stereotypy subscale, with a more favorable course for the continuation group was found (p=0.003).The Clinical Global Impression Scale-Improvement (CGI-I) (33)) showed a non-significant worsening in discontinuation group after 24 weeks (p>0.05).

Notably, discontinuation resulted in significant improvements in weight (p=0.046), waist circumference (p=0.012), BMI (p=0.030), systolic blood pressure (p=0.005) prolactin levels (p=0.007), and testosterone levels (p=0.048).

Overall, these results underline the need for regular reassessment of the indication for antipsychotic treatment and the fact that, contrary to what has been described for acute episodes, long-term treatment has not been shown to be effective. Despite the small number of patients, it also demonstrates the feasibility of deprescribing, which is a concern in clinical practice.

#### 3.1.4 Efficacy of Aripiprazole

The use of aripiprazole (mean dose, 9.8 mg/day) to manage irritability in individuals with Fragile X Syndrome (FXS) aged 6–25 years was examined in a prospective open-label study involving 12 participants (29). Subjects were considered treatment responders based on a CGI-I score of 1 or 2 and a ≥ 25% improvement of the ABC-I score. The study reported significant improvements in irritability, aggression, self-injury, and severe tantrums, in 10 out of the 12 subjects, as assessed using CGI-I (mean at endpoint: 1.6 ± 0.9) and ABC-I (a 72% decline from 25.3 at baseline to 7.1 at endpoint (p<0.001)).

Secondary outcomes, such as the ABC-Hyperactivity subscale (ABC-H), CGI-Severity (CGI-S) and Social Responsiveness Scale (SRS) (38), also showed significant improvements with treatment. Mean ABC-H scores declined by 51% from baseline to endpoint (p<0.001). Mean CGI-S scores decreased from 4.5 at baseline to 3.5 at endpoint (p=0.008). Total raw SRS scores improved by 28%, with a mean reduction from 124.5 at baseline to 90.1 at endpoint (p<0.001).

Adverse reactions were generally mild, with the most frequent symptoms being tiredness, nausea/vomiting, drooling, and insomnia. Two subjects withdrew from the study due to adverse reactions.

#### 3.1.5 Typical antipsychotic drugs

In a blinded discontinuation study, the effects of zuclopenthixol on aggressive behavior were assessed in 49 adults with mild to moderate ID (30). After a 6-week open treatment, 39 responders were randomized to either continue zuclopenthixol (n=19) or switch to a placebo (n=20) for up to 12 weeks. The zuclopenthixol group showed significantly lower aggressive behavior, as indicated by outcomes observed by external raters on the Modified Overt Aggression Scale (MOAS), with 37% (n=7) responders compared to 5% (n=1) in the placebo group. Kaplan–Meier estimates also indicated a significant difference in responder rates. Adverse events and withdrawal symptoms were similar between groups.

In a second study (31) they converted the short-term 12-week withdrawal trial into a 2-year open-label study with zuclopenthixol (2–20 mg/day). Out of 39 initial patients, 31 continued the treatment, with 21 remaining on medication after two years. Patients continuing treatment (CT) showed significant improvements in the MOAS, the Disability Assessment Schedule (DAS) (39), and the Clinical Global Impression (CGI) scale compared to the 10 drop-outs. The CT group had a three-point improvement in DAS scores, though some problematic behavior persisted. Doses, of 6 mg/day were considered the more appropriate, being effective and contributed to better tolerability and a lower rate of adverse events.

Early responders particularly benefited from ongoing treatment Zuclopenthixol.

Both studies (30, 31) supported the long-term efficacy and safety of zuclopenthixol, highlighting that early response predicted better outcomes.

### 3.2 Overview of Symptom-Specific Efficacy and Deprescribing Evidence

**Figure 1:**
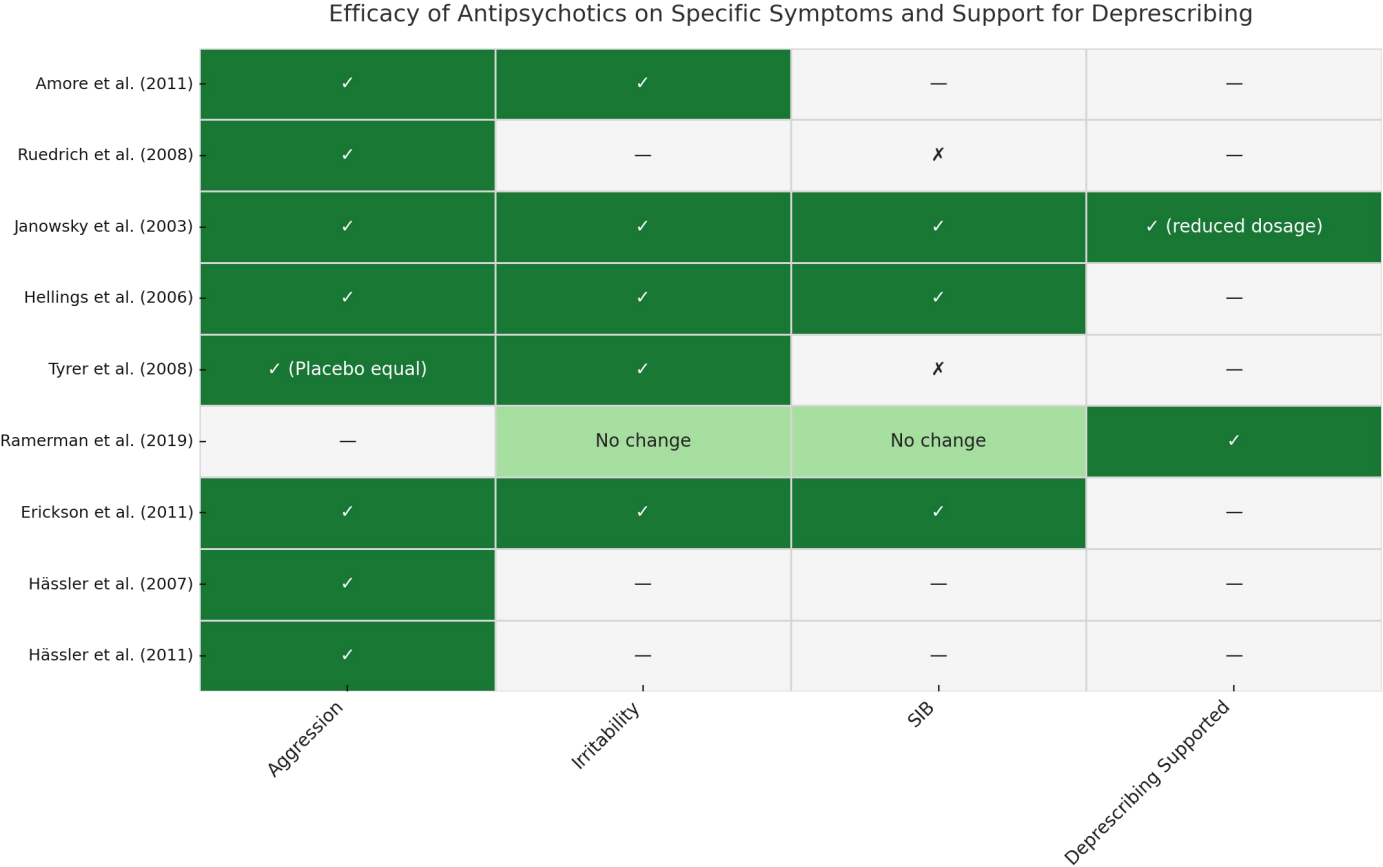
Efficacy of antipsychotics on specific symptoms and support for deprescribing.

To enhance the clarity of our findings, we compiled a synthesis of symptom-specific outcomes and deprescribing evidence across all included studies. A visual summary is provided in **Figure 1**, while detailed results are reported in **Supplementary Table 4**. This overview illustrates the heterogeneous responses to different antipsychotics across behavioral domains—such as aggression, irritability, and SIBs—and underscores the importance of personalized treatment planning.

This figure summarizes symptom-specific outcomes and deprescribing evidence from each included study. Rows correspond to individual studies, and columns represent the evaluated domains: aggression, irritability, SIBs, and support for deprescribing. ✓= Improvement observed in the specific domain; ✗ = No significant improvement; — = Not assessed or not reported; “No change” = No difference observed between intervention and control groups; “(Placebo equal)” = Antipsychotic showed similar effects to placebo; “(reduced dosage)” = Deprescribing supported through dose reduction rather than full discontinuation. Abbreviations: SIB = Self-Injurious Behavior; ID = Intellectual Disability. For detailed study characteristics and outcomes, see **Supplementary Table 4**.

## 4 Discussion

As anticipated, significant gaps remain in the literature regarding the efficacy and safety of antipsychotics drugs to treat BC in people with ID. By applying our criteria, we were only able to select a small number of studies, all of which included a small number of subjects (346 subjects all together, maximum 86 for the largest). Furthermore, the formal risk of bias assessment revealed that, although most RCTs were of moderate to high methodological quality, several observational studies presented moderate to serious concerns—particularly regarding confounding and participant selection. These limitations, detailed in **Supplementary Table 3**, underscore the fragility of the current evidence base. For this reason, a meta-analysis was not conducted. The small number of eligible studies, their heterogeneous designs, and the variability in outcome measures, interventions, and follow-up durations precluded meaningful quantitative synthesis. It is also worth noting that we deliberately excluded the term “autism spectrum disorder” from our search strategy, as previously explained in the introduction. While this decision likely limited the number of retrieved studies—as suggested by findings from a recent systematic review and meta-analysis by Deb et al. (40)—it was necessary to maintain population specificity and avoid the heterogeneity introduced by overlapping but distinct neurodevelopmental profiles. Nevertheless, there are notable similarities between our findings and those reported by Deb et al., particularly the lack of strong evidence supporting the use of antipsychotics to treat BC in both ASD and ID populations.

In their study, Deb et al. found some evidence for risperidone (14 RCTs) and preliminary evidence for aripiprazole (5 RCTs) to significantly reduces BC in children with ASD. They found no evidence for adults ASD population. However, our findings do not support a similar level of evidence and show mixed results regarding the efficacy of risperidone in ID population and no conclusive results regarding aripiprazole. Specifically, only one study (28) demonstrated some efficacy of long-term, low-dose risperidone in reducing irritability in children, adolescents, and adults with ID. Conversely, another study (26) reported no significant difference between risperidone and placebo in reducing BC.

Regarding adverse effects of antipsychotic treatment, Deb et al. reported a higher incidence of side effects such as weight gain and sedation in ASD populations. These findings are consistent with our observations in individuals with ID.

Overall, the studies presented showed that atypical antipsychotics, which are currently still preferred, can be just as effective as typical antipsychotics in this off-label use. Risperidone was the compound for which we identified the most data. In our selection, olanzapine was next, while aripiprazole was only studied in one small open-label trial in people with FXS. It should be noted that in the study of Deb et al., on subjects suffering from ASD, aripiprazole was involved in 5 RCTs, 4 of which were positive versus placebo for treating BC. The presence of an ID was only specified in one of the studies and was 63% of the included people. Regarding typical antipsychotic drug we only found data on low dose zuclopenthixol in 40 people (30, 31) as well as negative results with haloperidol in 30 people (26).

Although commonly accepted as effective, it is interesting to note that the largest study, a randomized controlled trial, showed no effect of risperidone compared to placebo for treating aggressive BC, after one month of treatment. In fact, a clear reduction in aggressive behaviors was reported in the 3 groups, risperidone, haloperidol and placebo, during the 1st month of treatment, underscoring the magnitude of the placebo effect (26). This observation challenges the assumption that antipsychotic medication is always necessary to achieve behavioral improvement in this population. It suggests that non-specific factors, such as increased attention, structured environments, or caregiver expectations, may contribute meaningfully to short-term outcomes. These insights reinforce the importance of reassessing treatment need regularly and considering non-pharmacological approaches as first-line interventions.

Another interesting point was that the efficacy of low doses of risperidone appeared to be equal to that of higher doses. High doses, which are associated with a higher risk of adverse effects, should therefore be avoided (28).

Regarding the types of BC, although there were some evidences in favor of atypical antipsychotics diminishing aggression and agitation, their impact on SIBs remains controversial. This might be explained by the sedative properties of some antipsychotics, for instance olanzapine, which may help mitigate aggressive behavior (41), but these sedative properties alone may not be enough to limit self-aggressive behaviors, as Ruedrich et al. (20) demonstrated in their study on risperidone and olanzapine. On the counterpart, Janowsky et al. and Erickson et al. (6, 29) reported positive outcomes on SIBs using olanzapine and aripiprazole respectively. These divergent findings underscore the variability in how atypical antipsychotics affect SIBs. Such inconsistency may be rooted in the multifactorial nature of SIBs, which involves dysregulation across several neurotransmitter systems—including dopaminergic, serotonergic, glutamatergic, and GABAergic pathways—as well as structural and functional abnormalities within fronto-limbic-striatal circuits (42). These neurobiological alterations can impair emotional regulation, behavioral inhibition, and reward processing, thereby contributing to the onset and persistence of SIB. This neurobiological and behavioral heterogeneity may help explain the mixed treatment outcomes observed across studies. It also highlights the need for improved phenotyping and the adoption of multimodal treatment strategies combining pharmacological and behavioral components.

Finally, the need to continue treatment in the medium to long term (>1 year) is being questioned. While some studies support long-term low-dose antipsychotic prescriptions (30, 31), others suggest the possibility of deprescribing without exacerbating BC. For example, Ramerman et al. (5) indicated that long-term treatment with risperidone can be withdrawn without increasing irritability. These findings advocate for considering deprescribing strategies, especially given the limited long-term efficacy and significant side effects associated with these medications, also opening windows for implementing non-pharmacological strategies which appear to be moderately effective (43).

Ramerman**’s** findings were further supported by various experiences, case reports and expert views on deprescribing psychotropic medication in ID population presenting challenging behaviors. A qualitative study by de Kuijper et al. (44) suggested that withdrawing antipsychotics is feasible with careful planning and support, involving healthcare professionals, patients, and their families to ensure a successful process. Similarly, a systematic review on deprescribing psychotropic medication for BC in people with ID (45), highlighted the complexities and potential benefits of deprescription by analyzing 54 studies and identifying both positive outcomes and substantial barriers. They emphasized that deprescribing can lead to behavioral and health improvements, particularly when implemented within an interdisciplinary care model, but also warned of risks such as relapse, dyskinesias, and the need for re-prescribing. The authors stressed the importance of pre-planned deprescribing strategies, stakeholder involvement, shared decision-making, and regular monitoring to ensure successful and safe implementation. Key factors for success include the role of primary caregivers, staff knowledge and skills (46), and realistic expectations about psychotropic medications, which are often overestimated.

An example of antipsychotics deprescription’s strategies is the structured withdrawal programme implemented by Shankar et al. (13). This program was enforced in Cornwall, UK and involved multiple steps and stakeholder engagement. Meetings were held with patients, carers, and professionals to discuss the withdrawal process, including potential risks and benefits. An oversight committee coordinated the program, integrating input from primary and secondary care, and facilitating regular Multi-Disciplinary Team discussions for case reviews. Specific tools were developed to assess and visualize risks and patient outcomes. Gradual dose reductions of 10-25% every 6–8 weeks were performed. A follow-up plan was established for each patient, with contingency strategies to manage any adverse effects during and after the withdrawal process. Results of their study show that with this structured approach, it was possible to completely withdraw antipsychotics in 46.5% of adults with ID and reduce the dose by over 50% in an additional 11.3% with no significant changes in the challenging behaviors.

These findings support the potential for wider implementation of structured withdrawal programmes to reduce inappropriate prescriptions on antipsychotics and long-term side effects, thereby improving the overall quality of life for individuals with ID.

One useful tool to guide clinicians in deprescription might be the Tool for Optimizing Prescription in Intellectual Disability (TOP-ID) (47). TOP-ID is a structured prescription and deprescription guide developed specifically for adults with ID. It aims to address common clinical situations such as pain management, gastrointestinal disorders, sleep disorders, and BC, providing a systematic approach to optimize medication use and minimize adverse effects. TOP-ID was developed using a four-step consensus-based process that included a review of the literature, semi-structured interviews, and a two-round Delphi process with 18 experts from various medical fields. This rigorous process ensured that the tool is based on both clinical expertise and the best available evidence. TOP-ID holds promise for clinical practice by providing a valuable tool for clinicians to make informed decisions about prescribing and deprescribing for this vulnerable population.

Furthermore, given the scarce evidence on the efficacy of antipsychotics, especially for long-term use, the importance of incorporating non-pharmacological methods cannot be overstated. These include behavioral therapies (e.g., positive behavioral support (PBS) or adapted dialectical behavior therapy (DBT)), environmental modifications, and caregiver training. For instance, a longitudinal study by Brown et al. demonstrated that adapted DBT can effectively reduce challenging behaviors in adults with intellectual and developmental disabilities (48) Similarly, recent evidence from a multicenter trial by Bruinsma et al. (49) suggests that PBS delivered by trained staff can improve quality of life among individuals with more severe CB or lower levels of intellectual functioning. Integrating both pharmacological and non-pharmacological interventions in a holistic approach may prove more effective in managing BC in individuals with ID (50, 51). Such comprehensive strategies, which include environmental modifications and behavioral therapies, could potentially provide more sustainable outcomes for individuals with ID, thereby ensuring a more balanced and effective management of their needs.

## 5 Limitations

This review has several limitations, including the small sample sizes of the included studies and significant heterogeneity across various factors, such as the type of antipsychotic used, dosage, duration of treatment, patient comorbidities, concomitant medications, underlying causes of intellectual disability, severity of ID, types of BC, age of patients, assessment scales, and study settings. These variations complicate the ability to generalize findings and make direct comparisons, thus limiting the overall applicability of the results. Additionally, given the considerable timespan covered by the included studies (1990–2024), it is important to acknowledge that both the definitions of ID and the pharmacological and non-pharmacological interventions have evolved substantially over time. This temporal variability represents an additional limitation, as it may affect the interpretation and comparability of findings across decades.

## 6 Conclusions and perspectives

This review underscores the lack of consistent results on the use of antipsychotics for managing BC in ID population in contrast to the observed clinical practices, relying often on medication.

The place of antipsychotic drugs in the care of BC has therefore to be specified.

There is no doubt that medication can be useful in behavioral crisis, but it is essential to think systematically, and repeatedly, about the medical and environmental causes of BC and to use non-pharmacological measures to prevent BC before using medication. Another important aspect is to assess carefully concomitant somatic and psychiatric disorders and to treat them.

Tools like TOP-ID can guide clinicians in this process by systematizing the approach and offering a step-by-step path that leads to more informed and patient-centered decisions, thereby minimizing the long-term use of antipsychotics and their associated side effects. Antipsychotics should, in fact, only be prescribed for a limited duration, unless justified by clinical response and ongoing need. This recommendation aligns with current international guidelines and reflects the principle that antipsychotics in individuals with ID should always be used within a framework of regular, structured reassessment of both indication and effectiveness.

Efforts should also be focused on implementation of deprescribing measures, widely described in the literature but sometimes difficult to set-up in everyday practice.

Future research should prioritize well-designed naturalistic studies and longitudinal observational designs to strengthen the evidence base for the use of antipsychotics in individuals with ID. These studies should focus on optimizing dosing strategies, exploring the long-term impact of these medications, and investigating additional pharmacological and non-pharmacological options. Despite the inherent challenges in conducting such studies within the ID population, the frequent use of antipsychotics in clinical practice makes it vital to equip clinicians with appropriate strategies and tools to optimize both the prescription and deprescription of psychotropic medications for challenging behavior. Such an approach can help tailor treatments to individual needs, minimize adverse effects, and ensure consistent monitoring, thus standardizing care across various clinical settings.

## Data Availability

The original contributions presented in the study are included in the article/**Supplementary Material**. Further inquiries can be directed to the corresponding author/s.

## Supplementary material

Supplementary Table 2 PRISMA (Preferred Reporting Items for Systematic Reviews and Meta-Analyses) flow diagram.

## References

[1] Maulik PK Mascarenhas MN Mathers CD Dua T Saxena S . Prevalence of intellectual disability: a meta-analysis of population-based studies. Res Dev Disabil. (2011) 32:419–36. doi: 10.1016/j.ridd.2010.12.018, PMID: 21236634

[2] Lee K Cascella M Marwaha R . Intellectual disability. In: StatPearls. StatPearls Publishing, Treasure Island (FL (2024). Available online at: http://www.ncbi.nlm.nih.gov/books/NBK547654/. 31613434

[3] Schofield D Shrestha R Tan O Lim K Rajkumar R West S . The healthcare and societal costs of familial intellectual disability. Int J Environ Res Public Health. (2024) 21:299. doi: 10.3390/ijerph21030299, PMID: 38541298 PMC10970490

[4] Ali A Blickwedel J Hassiotis A . Interventions for challenging behavior in intellectual disability. Adv Psychiatr Treat. (2014) 20:184–92. doi: 10.1192/apt.bp.113.011577

[5] Ramerman L de Kuijper G Scheers T Vink M Vrijmoeth P Hoekstra PJ . Is risperidone effective in reducing challenging behaviors in individuals with intellectual disabilities after 1 year or longer use? A placebo-controlled, randomized, double-blind discontinuation study. J Intellect Disabil Res. (2019) 63:418–28. doi: 10.1111/jir.12584, PMID: 30609152

[6] Janowsky DS Barnhill LJ Davis JM . Olanzapine for self-injurious, aggressive, and disruptive behaviors in intellectually disabled adults: a retrospective, open-label, naturalistic trial. J Clin Psychiatry. (2003) 64:1258–65. doi: 10.4088/JCP.v64n1018, PMID: 14658977

[7] Song M Ware R Doan TN Harley D . Psychotropic medication use in adults with intellectual disability in Queensland, Australia, from 1999 to 2015: a cohort study. J Intellect Disabil Res. (2020) 64:45–56. doi: 10.1111/jir.12685, PMID: 31478300

[8] Deb S Unwin G Deb T . Characteristics and the trajectory of psychotropic medication use in general and antipsychotics in particular among adults with an intellectual disability who exhibit aggressive behavior. J Intellect Disabil Res JIDR. (2015) 59:11–25. doi: 10.1111/jir.12119, PMID: 24450426

[9] Deb S Kwok H Bertelli M Salvador Carulla L Bradley E Torr J . International guide to prescribing psychotropic medication for the management of problem behaviors in adults with intellectual disabilities. World Psychiatry. (2009) 8:181–6. doi: 10.1002/j.2051-5545.2009.tb00248.x, PMID: 19812757 PMC2758582

[10] Quality statement 11: Use of medication | Learning disability: behavior that challenges | Quality standards | NICE. NICE. (2015). Available online at: https://www.nice.org.uk/guidance/qs101/chapter/quality-statement-11-use-of-medication (Accessed July 21, 2025).

[11] Quality statement 12: Review of medication | Learning disability: behavior that challenges | Quality standards | NICE. NICE. (2015). Available online at: https://www.nice.org.uk/guidance/qs101/chapter/quality-statement-12-review-of-medication (Accessed July 21, 2025).

[12] Sullivan WF Diepstra H Heng J Ally S Bradley E Casson I . Primary care of adults with intellectual and developmental disabilities. Can Fam Physician. (2018) 64:254–79. PMC5897068 29650602

[13] Shankar R Wilcock M Deb S Goodey R Corson E Pretorius C . A structured programme to withdraw antipsychotics among adults with intellectual disabilities: The Cornwall experience. J Appl Res Intellect Disabil. (2019) 32:1389–400. doi: 10.1111/jar.12635, PMID: 31192534

[14] O’Dwyer C McCallion P Henman M McCarron M O’Leary E Burke E . Prevalence and patterns of antipsychotic use and their associations with mental health and problem behaviors among older adults with intellectual disabilities. J Appl Res Intellect Disabil. (2019) 32:981–93. doi: 10.1111/jar.12591, PMID: 31038275

[15] Tsiouris JA . Pharmacotherapy for aggressive behaviors in persons with intellectual disabilities: treatment or mistreatment? J Intellect Disabil Res. (2010) 54:1–16. doi: 10.1111/j.1365-2788.2009.01232.x, PMID: 20122096

[16] Lonchampt S Gerber F Aubry JM Desmeules J Kosel M Besson M . Prevalence of polypharmacy and inappropriate medication in adults with intellectual disabilities in a hospital setting in Switzerland. Front Psychiatry. (2021) 12:614825. doi: 10.3389/fpsyt.2021.614825, PMID: 34248693 PMC8267250

[17] McMahon M Hatton C Bowring DL Hardy C Preston NJ . The prevalence of potential drug-drug interactions in adults with intellectual disability. J Intellect Disabil Res. (2021) 65:930–40. doi: 10.1111/jir.12844, PMID: 33988262

[18] Campos-Jara R Martínez-Salazar C Campos-Jara C Fernández JM Martínez-García D Contreras-Osorio F . Pharmacological treatment for challenging behavior in adults with intellectual disability: Systematic review and meta-analysis. Rev Psiquiatr Salud Ment. (2023) 17:S1888–9891(23)00004-6. doi: 10.1016/j.rpsm.2023.01.003, PMID: 37839961

[19] Iffland M Livingstone N Jorgensen M Hazell P Gillies D . Pharmacological intervention for irritability, aggression, and self-injury in autism spectrum disorder (ASD). Cochrane Database Syst Rev. (2023) 10:CD011769. doi: 10.1002/14651858.CD011769.pub2, PMID: 37811711 PMC10561353

[20] Ruedrich SL Swales TP Rossvanes C Diana L Arkadiev V Lim K . Atypical antipsychotic medication improves aggression, but not self-injurious behavior, in adults with intellectual disabilities. J Intellect Disabil Res. (2008) 52:132–40. doi: 10.1111/j.1365-2788.2007.00981.x, PMID: 18197952

[21] Thurm A Farmer C Salzman E Lord C Bishop S . State of the field: differentiating intellectual disability from autism spectrum disorder. Front Psychiatry. (2019) 10. Available online at. doi: 10.3389/fpsyt.2019.00526, PMID: 31417436 PMC6683759

[22] Page MJ McKenzie JE Bossuyt PM Boutron I Hoffmann TC Mulrow CD . The PRISMA 2020 statement: An updated guideline for reporting systematic reviews. BMJ (Clinical Res Ed). (2021) 372:n71. doi: 10.1136/bmj.n71, PMID: 33782057 PMC8005924

[23] Amir-Behghadami M Janati A . Population, Intervention, Comparison, Outcomes and Study (PICOS) design as a framework to formulate eligibility criteria in systematic reviews. Emergency Med J. (2020) 37:387. doi: 10.1136/emermed-2020-209567, PMID: 32253195

[24] Sterne JAC Savović J Page MJ Elbers RG Blencowe NS Boutron I . RoB 2: a revised tool for assessing risk of bias in randomized trials. BMJ. (2019) 366:l4898. doi: 10.1136/bmj.l4898, PMID: 31462531

[25] Sterne JA Hernán MA Reeves BC Savović J Berkman ND Viswanathan M . ROBINS-I: a tool for assessing risk of bias in non-randomized studies of interventions. BMJ. (2016) 355:i4919. doi: 10.1136/bmj.i4919, PMID: 27733354 PMC5062054

[26] Tyrer P Oliver-Africano PC Ahmed Z Bouras N Cooray S Deb S . Risperidone, haloperidol, and placebo in the treatment of aggressive challenging behavior in patients with intellectual disability: a randomized controlled trial. Lancet. (2008) 371:57–63. doi: 10.1016/S0140-6736(08)60072-0, PMID: 18177776

[27] Amore M Bertelli M Villani D Tamborini S Rossi M . Olanzapine vs. risperidone in treating aggressive behaviors in adults with intellectual disability: a single blind study. J Intellect Disabil Res. (2011) 55:210–8. doi: 10.1111/j.1365-2788.2010.01352.x, PMID: 21129058

[28] Hellings JA Zarcone JR Reese RM Valdovinos MG Marquis JG Fleming KK . A crossover study of risperidone in children, adolescents and adults with mental retardation. J Autism Dev Disord. (2006) 36:401–11. doi: 10.1007/s10803-006-0078-1, PMID: 16596465

[29] Erickson CA Stigler KA Wink LK Mullett JE Kohn A Posey DJ . A prospective open-label study of aripiprazole in fragile X syndrome. Psychopharmacol (Berl). (2011) 216:85–90. doi: 10.1007/s00213-011-2194-7, PMID: 21318565

[30] Haessler F Glaser T Beneke M Pap AF Bodenschatz R Reis O . Zuclopenthixol in adults with intellectual disabilities and aggressive behaviors: Discontinuation study. Br J Psychiatry. (2007) 190:447–8. doi: 10.1192/bjp.bp.105.016535, PMID: 17470962

[31] Häßler F Glaser T Reis O . Effects of zuclopenthixol on aggressive disruptive behavior in adults with mental retardation – A 2-year follow-up on a withdrawal study. Pharmacopsychiatry. (2011) 44:339–43. doi: 10.1055/s-0031-1291174, PMID: 21993867

[32] Yudofsky SC Silver JM Jackson W Endicott J Williams D . The Overt Aggression Scale for the objective rating of verbal and physical aggression. Am J Psychiatry. (1986) 143:35–9. doi: 10.1176/ajp.143.1.35, PMID: 3942284

[33] Busner J Targum SD . The clinical global impressions scale. Psychiatry Edgmont. (2007) 4:28–37. PMC2880930 20526405

[34] Aman MG Singh NN Stewart AW Field CJ . The aberrant behavior checklist: a behavior rating scale for the assessment of treatment effects. Am J Ment Defic. (1985) 89:485–91. 3993694

[35] Chukwujekwu DC Stanley PC . The Modified Overt Aggression Scale: how valid in this environment? Niger J Med J Natl Assoc Resid Dr Niger. (2008) 17:153–5. doi: 10.4314/njm.v17i2.37373, PMID: 18686830

[36] Ono Y . Factor validity and reliability for the Aberrant Behavior Checklist-Community in a Japanese population with mental retardation. Res Dev Disabil. (1996) 17:303–9. doi: 10.1016/0891-4222(96)00015-7, PMID: 8827840

[37] Lingjaerde O Ahlfors UG Bech P Dencker SJ Elgen K . The UKU side effect rating scale. A new comprehensive rating scale for psychotropic drugs and a cross-sectional study of side effects in neuroleptic-treated patients. Acta Psychiatr Scand Suppl. (1987) 334:1–100. doi: 10.1111/j.1600-0447.1987.tb10566.x, PMID: 2887090

[38] Chan W Smith LE Hong J Greenberg JS Mailick MR . Validating the social responsiveness scale for adults with autism. Autism Res. (2017) 10:1663–71. doi: 10.1002/aur.1813, PMID: 28639377 PMC5648615

[39] Tsakanikos E Underwood L Sturmey P Bouras N McCarthy J . Psychometric properties of the Disability Assessment Schedule (DAS) for behavior problems: an independent investigation. Res Dev Disabil. (2011) 32:653–8. doi: 10.1016/j.ridd.2010.12.004, PMID: 21208774

[40] Deb S Roy M Limbu B Akrout Brizard B Murugan M Roy A . Randomised controlled trials of antipsychotics for people with autism spectrum disorder: a systematic review and a meta-analysis. Psychol Med. (2023) 53:7964–72. doi: 10.1017/S003329172300212X, PMID: 37539448

[41] Zareifopoulos N Panayiotakopoulos G . Treatment options for acute agitation in psychiatric patients: theoretical and empirical evidence. Cureus. (2019) 11:e6152. doi: 10.7759/cureus.6152, PMID: 31890361 PMC6913952

[42] Zhang K Ibrahim GM Venetucci Gouveia F . Molecular pathways, neural circuits and emerging therapies for self-injurious behavior. Int J Mol Sci. (2025) 26:1938. doi: 10.3390/ijms26051938, PMID: 40076564 PMC11900092

[43] Bruinsma E van den Hoofdakker BJ Groenman AP Hoekstra PJ de Kuijper GM Klaver M . Non-pharmacological interventions for challenging behaviors of adults with intellectual disabilities: A meta-analysis. J Intellect Disabil Res. (2020) 64:561–78. doi: 10.1111/jir.12736, PMID: 32558050 PMC7384078

[44] de Kuijper G de Haan J Deb S Shankar R . Withdrawing antipsychotics for challenging behaviors in adults with intellectual disabilities: experiences and views of experts by experience. Int J Environ Res Public Health. (2022) 19:15637. doi: 10.3390/ijerph192315637, PMID: 36497711 PMC9736624

[45] Adams D Hastings RP Maidment I Shah C Langdon PE . Deprescribing psychotropic medicines for behaviors that challenge in people with intellectual disabilities: A systematic review. BMC Psychiatry. (2023) 23:202. doi: 10.1186/s12888-022-04479-w, PMID: 36978032 PMC10044393

[46] Kleijwegt B Pruijssers A de Jong-Bakker L de Haan K van Os-Medendorp H van Meijel B . Support staff’s perceptions of discontinuing antipsychotics in people with intellectual disabilities in residential care: A mixed-method study. J Appl Res Intellect Disabil JARID. (2019) 32:861–70. doi: 10.1111/jar.12577, PMID: 30790388 PMC6850344

[47] Lonchampt S Gerber F Aubry JM Desmeules J Besson M Kosel M . TOP-ID: a Delphi technique-guided development of a prescription and deprescription tool for adults with intellectual disabilities. BMJ Open. (2020) 10:e039208. doi: 10.1136/bmjopen-2020-039208, PMID: 33148748 PMC7643515

[48] Brown JF Brown MZ Dibiasio P . Treating individuals with intellectual disabilities and challenging behaviors with adapted dialectical behavior therapy. J Ment Health Res Intellect Disabil. (2013) 6:280–303. doi: 10.1080/19315864.2012.700684, PMID: 23914278 PMC3725667

[49] Bruinsma E van den Hoofdakker BJ Hoekstra PJ de Kuijper GM de Bildt AA . Effects of positive behavior support delivered by direct staff on challenging behaviors and quality of life of adults with intellectual disabilities: A multicentre cluster-controlled trial. J Appl Res Intellect Disabil JARID. (2024) 37:e13164. doi: 10.1111/jar.13164, PMID: 37899656

[50] Groves L Jones C Welham A Hamilton A Liew A Richards C . Non-pharmacological and pharmacological interventions for the reduction or prevention of topographies of behaviors that challenge in people with intellectual disabilities: a systematic review and meta-analysis of randomized controlled trials. Lancet Psychiatry. (2023) 10:682–92. doi: 10.1016/S2215-0366(23)00197-9, PMID: 37595996

[51] Ji NY Findling RL . Pharmacotherapy for mental health problems in people with intellectual disability. Curr Opin Psychiatry. (2016) 29:103–25. doi: 10.1097/YCO.0000000000000233, PMID: 26779860

