# Supplementary material for: Antipsychotic medication for behaviors that challenge in individuals with intellectual disabilities: a clinically informed review
Source: Front Psychiatry. 2025 Jul 28;16:1609408. doi: 10.3389/fpsyt.2025.1609408 (PMC12336111; doi:10.3389/fpsyt.2025.1609408)
Supplement: Supplementary file 1 [file Table1.docx]

**Supplementary Table 1**. Search strategy according to the Population, Intervention, Comparison, Outcomes and Study Design (PICOS) model

| Parameter | Inclusion criteria | Exclusion criteria |
| --- | --- | --- |
| Population | - Patients with intellectual disabilities (ID) including fragile X syndrome (FXS) (DSM/ICD diagnosis) presenting challenging behaviors | - Patients with neurovascular or neurodegenerative or age-related cognitive impairment - Patients without ID nor FXS |
| Interventions | - Any antipsychotic medication | - No antipsychotic medication |
| Comparison | - Any comparison | NA |
| Outcomes | - Changes in challenging behaviors | NA |
| Study design model | Observational cross-sectional or longitudinal studies, or randomized controlled trials | Case reports, abstracts, conference presentations, case series including <10 patients, reviews, metanalyses and systematic reviews |
