# Supplementary material for: Antipsychotic medication for behaviors that challenge in individuals with intellectual disabilities: a clinically informed review
Source: Front Psychiatry. 2025 Jul 28;16:1609408. doi: 10.3389/fpsyt.2025.1609408 (PMC12336111; doi:10.3389/fpsyt.2025.1609408)
Supplement: Supplementary Table 2 — PRISMA (Preferred Reporting Items for Systematic Reviews and Meta-Analyses) flow diagram. [file Table2.docx]

**Identification of studies via database**

Records identified from:

Pubmed

**Identification**

Records screened for title and abstract:

(n = 221)

Records excluded:

(n = 199 )

**Screening**

Reports assessed for eligibility:

(n = 22 )

Reports excluded: 14

Wrong study design (n = 8 )

Wrong intervention (n = 2 )

Wrong population (n = 4)

Studies included in review

(n = 9 ): 8 studies from the original search strategy and 1 additional study found consulting previous study bibliography.

**Included**

*From:*  Page MJ, McKenzie JE, Bossuyt PM, Boutron I, Hoffmann TC, Mulrow CD, et al. The PRISMA 2020 statement: an updated guideline for reporting systematic reviews. BMJ 2021;372:n71. doi: 10.1136/bmj.n71
